# Parenting-Related Factors Associated With Depression and Anxiety: A Systematic Review and Meta-Analysis

**DOI:** 10.7759/cureus.103388

**Published:** 2026-02-10

**Authors:** Mohammed M AL-Thiab, Majed M Alshehri, Abdulrahman S AlQumayzi, Amjad K Abumilha, Abdulmajeed M Alzahrani, Sultan M Alsharef, Salem K Almasar, Abdulrahim S Alamri

**Affiliations:** 1 Psychiatry, Armed Forces Hospital in Southern Region, Khamis Mushayt, SAU; 2 Psychiatry, Armed Forces Hospital In Southern Region, Khamis Mushayt, SAU; 3 Psychiatry, Abha Psychiatric Hospital, Aseer, SAU; 4 Psychiatry, Riyadh Joint Program, Riyadh, SAU; 5 Psychiatry, Tabuk Psychiatric Hospital, Tabuk, SAU; 6 General Practice, Aseer Health Cluster, Aseer, SAU

**Keywords:** aging, anxiety, childlessness, depression, meta-analysis, older adults, parenthood, systematic review

## Abstract

Aging populations and the increasing burden of depression and anxiety highlight the need to identify modifiable psychosocial determinants. Parenting-related factors may influence depression and anxiety risk and severity, but findings remain heterogeneous. We conducted a PRISMA-based systematic review and meta-analysis examining associations between parenting-related factors and depression and anxiety outcomes. MEDLINE, Embase, PsycINFO, Scopus, and CINAHL were searched from inception through 2025. Observational studies reporting quantitative associations were included, and effect estimates were synthesized using random-effects meta-analysis where appropriate. Risk of bias was assessed using a modified Joanna Briggs Institute checklist. Fifty-two studies were included. Quantitative synthesis indicated significant associations for several parenting-related factors. In meta-analyses, parental self-efficacy showed a large association with depression (d = 0.96), parenting stress showed strong associations with depression (d = 0.75) and anxiety (d = 0.74), and parent-child relationship quality and warmth/support were associated with lower depression. Findings emphasize that depression and anxiety are not driven by parental status alone but are associated with relational and psychosocial parenting-related dimensions. These results support targeting modifiable parenting-related mechanisms (e.g., stress, self-efficacy, relationship quality) in prevention and intervention strategies.

## Introduction and background

The global challenge of late-life mental health

The global population aged 60 years and older is projected to increase from approximately 1.1 billion in 2022 to 2.1 billion by 2050, representing an almost two-fold rise [1]. In line with this demographic transformation, depression and anxiety are key factors leading to disability, morbidity, and medical use in later adulthood. Depressive disorders were ranked among the leading causes of years lived with disability amongst the aged world over, and anxiety disorders often find their way into the system, worsening functional impairments and health care demands [1]. Depression and anxiety are multifactorial conditions influenced by interacting biological and psychosocial determinants, including chronic medical illness, functional impairment, pain, trauma exposure, socioeconomic adversity, relationship stressors, and social isolation. Parenting-related exposures, therefore, represent only one component within a broader etiological framework, and observed associations should be interpreted in the context of these interacting risk factors.

Recent surveillance statistics in high-income nations indicate that about 7-10% of adults aged 60 years and older have clinically significant symptoms of depression, and even a much higher proportion of adults have subthreshold symptoms [2]. Moderate-to-severe depressive symptoms were reported in 8.7% of adults aged 60 years and above in the United States in 2021-2023, and the trend is increasing compared to the previous decade [3]. Although less commonly diagnosed in the elderly, anxiety disorders are common and underappreciated, thus leading to poorer quality of life, higher risk of falls, cognitive disruption, and unnecessary mortality [4]. It is linked to both depression and anxiety in later life, which is linked to the increased all-cause mortality, the worse management of chronic disease, and the institutionalization, as well as the significantly increased healthcare expenditure [5]. It is thus a public health necessity to identify some modifiable social determinants and protective factors.

The evolving social landscape of aging

At the same time, there has been a radical shift in the social context of aging. The decline in fertility, postponement of childbearing, and increase in divorce rates, and a dynamic approach to marriage and reproduction have led to a growing proportion of both voluntary and involuntary childhoods. Over 15-25% of adults joining older adulthood in high-income nations do so without children, and it is projected that there will be more in future generations to come [6]. These changes in demography are of concern due to the loss of traditional family forms of support that have historically softened psychological distress later in life.

Adult children may often be a major source of emotional support and instrumental aid as well as social inclusion on the part of older adults, especially in situations where there are restricted formal long-term systems of care. On the other hand, childlessness can make a person more vulnerable to loneliness and social isolation, which are risk factors of late-life depression and anxiety [7]. Nonetheless, parenthood does not consistently lead to support; geographic dispersion, negative relationships, or caregiving reversals might mitigate or overturn the possible mental health gains. It is therefore crucial to understand the way parenthood works in contemporary aging societies.

Theoretical underpinnings

A number of theoretical frameworks form a basis for making hypotheses on the relationship between parenthood and mental health in adulthood. According to the Socioemotional Selectivity Theory, when the perceived time horizons become shorter, older people focus on the relationships that are emotionally significant to them (the most frequent being close family members, including children), which leads to better emotional management and psychological well-being [8].

The Convoy Model of Social Relations is a concept of social ties as a dynamic network that is present throughout the life course, and the children usually take a central, long-term role, which supports and brings permanence in old age [9]. Role theory also implies that parenthood is a social role of long-term identity, purpose, and normative expectations, which can prevent depression, as the role provides meaning and social integration following retirement or widowhood [10].

Conversely, the Stress Process Model indicates the possibility of stress on the role of parents, especially in cases where the adult children are indicative of unemployment, illness, or dependency, or when the parents are involved in the care of the grandchildren or dependent children [11]. Parenthood can then be either a role-enhancing or role-straining process based on the contextual and relational variables.

The ambiguous evidence

There is incongruent empirical data on late-life mental health and parenthood. There are some studies that show depressive symptom burden is lower in parents than in childless counterparts, and some that show no or even negative correlations. According to recent cohort studies in the U.S., it is indicated that changes with adjustment for marital status and socioeconomic status would show a negligible difference and gender-specific changes [12]. There is also a great deal of evidence that is cross-national; to wit, large multinational studies have found protection relationships in some Asian settings and none in Western Europe/Northern America, which is why cultural norms and welfare regimes are a concern [13]. Studies based on longitudinal registers have even reported a slightly reduced depression risk among the childless elderly, which may be due to the selection or stressor exposure, which is different [14].

Rationale and research gaps

Despite increasing interest, literature is heterogeneous in terms of methodological heterogeneity, such as inconsistent definitions of parenthood, different measurements of depression and anxiety, cross-sectional study designs that are susceptible to reversal causation, and poor control of confounding by marital status, health, and socioeconomic position. Notably, no meta-analysis has been done to quantitatively synthesize such results across cultures or to look at such moderators as gender, quality of relationship, or contact frequency. In particular, the outcomes of anxiety are underrepresented. These are the gaps that restrict the opportunity to make definite conclusions about whether parenthood is one of the protective factors in later life.

Research questions and objectives

This systematic review and meta-analysis aimed to synthesize quantitative evidence on parenting-related factors associated with depression and anxiety outcomes.

Primary Research Question

Which parenting-related factors are associated with depression and/or anxiety outcomes?

Secondary Research Questions

Do these associations vary according to (1) child developmental stage or age group; (2) informant type (parent-, child-, or clinician-reported); (3) cultural or regional context; (4) study design and adjustment level; and (5) specific domains of parenting-related exposure (e.g., parenting stress, parental self-efficacy, warmth/support, conflict, discipline, and overinvolvement)?

## Review

Methods

Search strategy, inclusion criteria, outcomes, and methods of data synthesis were pre-registered on PROSPERO (CRD420261287266). This review followed the Preferred Reporting Items of Systematic Reviews and meta-analyses (PRISMA) statement [15].

Search Strategy

A search of Embase, OVID MEDLINE, PsycINFO, Scopus, and CINAHL was carried out on 23 November 2021 and updated on 24 November 2022 to 2025. Search strings were (1) parents, (2) anxiety/depression, and (3) parenting. There were no restrictions on the date or language. Included and relevant reviews were searched through reference lists. The complete search strategy for all databases is provided in the Appendix (Table 3).

Study Selection

The inclusion criteria included (a) peer-reviewed studies, (b) the comparison of parents with current depression and/or anxiety problems and non-clinical controls, (c) measurement of mental health using diagnostic interview, professional diagnosis, or validated self-report measure above a clinical cut-off, (d) at least one modifiable parental factor, and (e) studies with children aged 2 to 18.99 years.

We filtered out studies that dealt exclusively with past mental health, non-English languages, composite parental variables, or studies that had a different child health status between groups.

Screening and Data Extraction

Duplicates were eliminated with the help of Covidence software. Researchers screened titles/abstracts and full texts independently. A standardized form was used to extract data on study characteristics, participant demographics, mental health-based criteria, parental factors, and outcome statistics.

Parental Factors Categorization

Extracted parental factors were categorized into 14 conceptually similar, modifiable categories according to prior systematic reviews [16,17]. Table 1 provides definitions, with category operationalizations informed by earlier parenting-factor syntheses and related sources [18-22]. Parent-child conflict was included as a distinct relational domain [23]. Parent-child relationship quality and related constructs were defined using prior syntheses and sources [24,25]. Parental self-efficacy was treated as a distinct construct based on prior work [26]. This approach is consistent with prior syntheses examining parenthood-related depressive outcomes and mechanisms across studies [27]. Parenting stress, satisfaction, and physical discipline/abuse were defined using established sources [28-30]. Withdrawal-related patterns were included as a separate modifiable domain [31].

**Table 1:** Parental factors categorization

Parental Factor	Definition
Authoritarian Parenting	Strict control, demand for obedience, and harsh disciplinary practices [18-20].
Aversiveness	Hostility, criticism, verbal aggression, and parental rejection [17].
Encouraging Healthy Habits	Promotion of healthy eating, physical activity, adequate sleep, and appropriate screen time in children [21].
Monitoring	Parental knowledge of the child’s activities and whereabouts, reflecting appropriate supervision [16,22].
Overinvolvement	Interference with child autonomy through psychological control or overprotection [16].
Parent Child Conflict	Ongoing tension, disagreements, and arguments between parent and child [23].
Parent Child Relationship Quality	Perceived closeness, secure attachment, and quality of positive parent child interactions [24,25].
Parental Discipline	Effective and consistent behavior management and enforcement of rules [24].
Parental Self Efficacy	Parental confidence in the ability to perform parenting tasks effectively [26].
Parenting Stress	Psychological and emotional strain associated specifically with the parenting role [28].
Parenting Satisfaction	Feelings of enjoyment, fulfillment, and reward derived from parenting [29].
Physical Discipline or Abuse	Use of physical force ranging from corporal punishment to maltreatment [30].
Warmth and Support	Expressions of positive regard, affection, praise, and active involvement with the child [24].
Withdrawal	Parental disengagement, disinterest, or neglect of the child’s emotional and practical needs [17,31].

These categories included parent-child conflict as a distinct relational domain [23]. Withdrawal-related patterns were also included as a separate modifiable domain [31].

Risk of Bias Assessment

The checklist used to evaluate risk of bias was based on the Joanna Briggs Institute (JBI) Critical Appraisal Checklist of Case Control Studies [32]. The rating of each study was done by two researchers. A study was considered low risk when 70% or more of the criteria were met, moderate when 50-69%, and high when less than 50%.

Data Synthesis

A meta-analysis and narrative synthesis were conducted. The primary quantitative synthesis used random-effects meta-analysis where sufficient comparable data were available, and effect sizes could be expressed on a consistent scale (standardized mean difference, Cohen’s d). For domains where studies reported heterogeneous outcomes or insufficient data for pooling, evidence was summarized using structured narrative synthesis. Where applicable, weighted-z methods were used as a supplementary approach to integrate findings across studies when effect size harmonization was not feasible, with weighting by sample size [33].

Meta-Analysis Procedures

A meta-analysis was conducted when two or more independent samples provided unadjusted data that could be transformed into a standardized mean difference (Cohen's d). Comprehensive meta-analysis software employed random effects. Heterogeneity was measured using Q statistics and prediction intervals. Publication bias was evaluated using funnel plots and the Egger test with k = 3, and the trim-and-fill procedure was used to adjust for bias. Informant type, child age, country individualism index (IDV), and anxiety type were examined using subgroup analyses and meta-regressions when adequate data were available (k ≥ 3 per subgroup) [34].

Results

Study Selection

**Figure 1:**
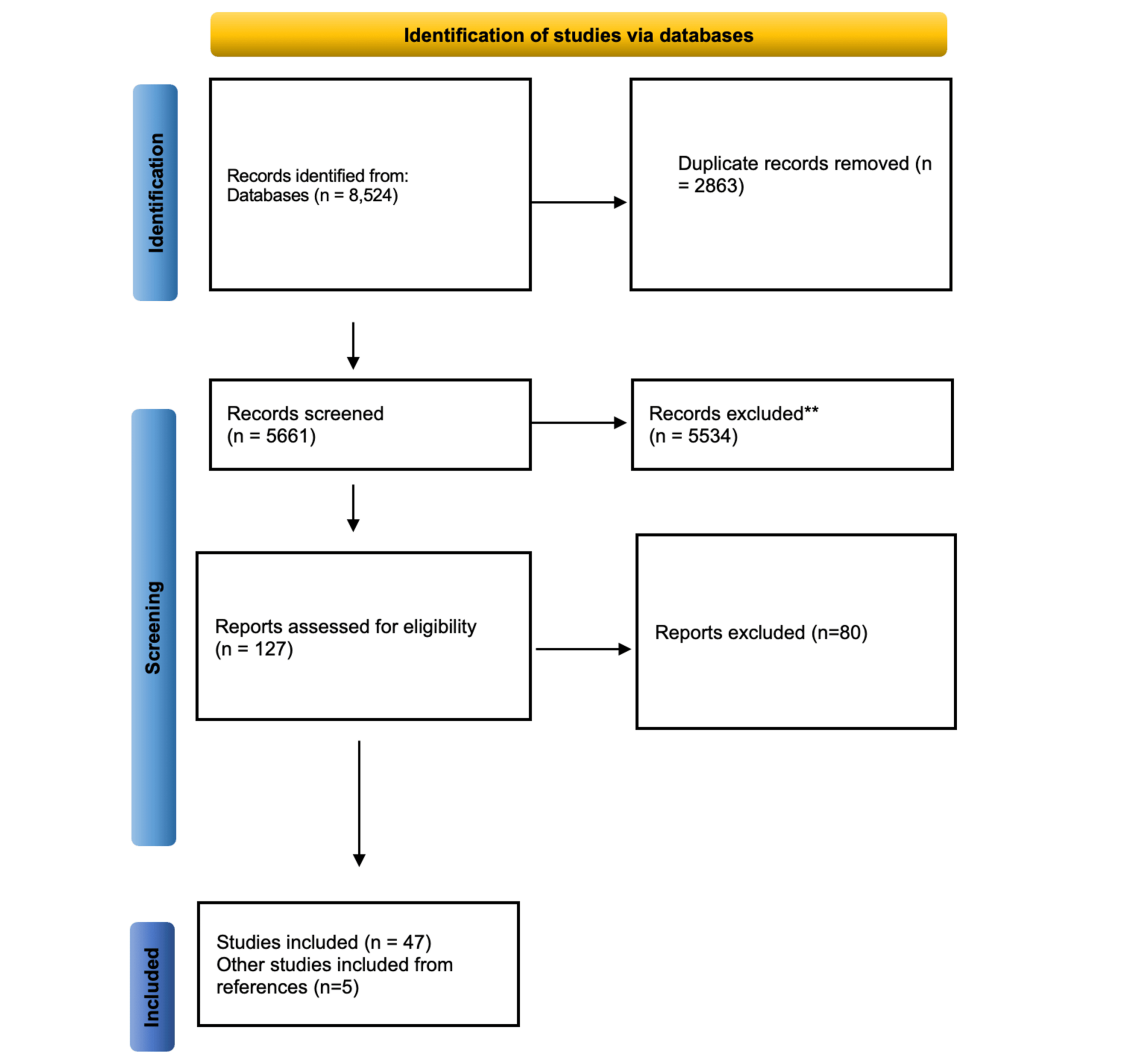
PRISMA flow diagram detailing the screening process.

The process of study selection was based on PRISMA guidelines [15], and is summarized in the PRISMA flow diagram (Figure 1). The number of duplicates removed was 41,075 titles and abstracts, after which 2,088 full-text articles were evaluated for eligibility. The final sample included 52 studies published between 1996 and 2024, with 17,657 participants. To conduct weighted-z analyses, 141 comparisons in 51 articles were used, and 118 comparisons in 44 papers were included in the meta-analyses [33].

Risk of Bias

The overall risk of bias of included studies was evaluated using systematic review etiology criteria [32]. Among the 51 studies included in the analyses, 28 (55%) were at low risk of bias, 21 (41%) were at moderate risk of bias, and 2 (4%) were at high risk of bias. The main constraints contributing to moderate risk were the predominance of cross-sectional designs (limiting causal inference) and the possibility of residual confounding by unmeasured factors.

Synthesis of Results

**Figure 2:**
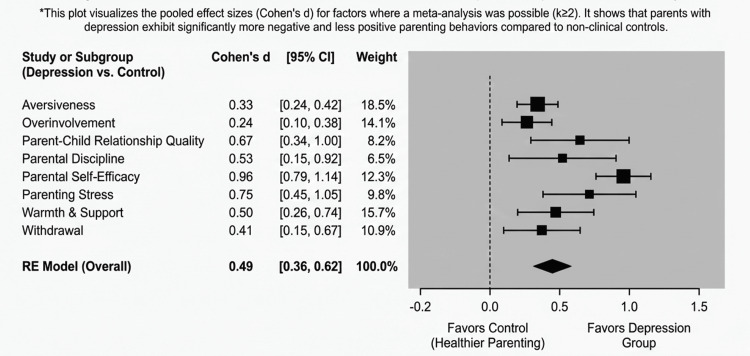
Forest plot of parental factors associated with depression (parents vs. control) This plot visualizes the pooled effect sizes (Cohen's d) for factors where a meta-analysis was possible (k≥2). It shows that parents with depression exhibit significantly more negative and less positive parenting behaviors compared to non-clinical controls.

**Figure 3:**
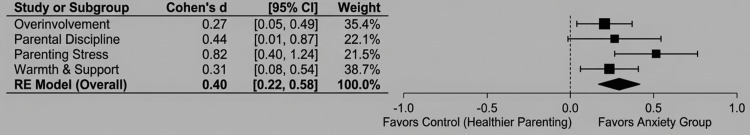
Forest plot of parental factors associated with anxiety (parents vs. control) This plot shows the pooled effects for anxiety. While the weighted-z analysis showed fewer significant factors, the meta-analysis quantifies the magnitude of differences where data were available.

Table 2 presents the summary of results for each parental factor. The random-effects meta-analysis results for depression are summarized in the forest plot (Appendix, Figure 2). The corresponding random-effects meta-analysis results for anxiety are summarized in the forest plot (Appendix, Figure 3). The case-by-case synthesis indicates that the relationship between parenthood and mental health depends on specific aspects of relationships and behavior. 

**Table 2:** Summary of findings from weighted z and meta-analyses Note: k equals the number of comparisons. d is the Cohen d. CI is abbreviated confidence interval. The factors that result significantly or have enough data are only displayed. Wtd z represents weighted z analysis as per Whitlock (2005). Meta is an indication of meta-analysis. When there is a dash, then there is not enough data to analyze. Statistical significance at p = .05 and p = .01.

Parental Factor	Depression vs. Control Wtd z (k)	p	Meta (k)	d [95% CI]	Anxiety vs. Control Wtd z (k)	p	Meta (k)	d [95% CI]	Comorbidity vs. Control Wtd z (k)	p	Meta (k)	d [95% CI]
Aversiveness	-13	<0.001	-10	0.33 [0.24, 0.42]	-10	0.264	-9	0.53 [0.20, 0.87]	-1	–	-1	–
Overinvolvement	-8	0.156	-7	0.24 [0.10, 0.38]	-8	0.152	-7	0.32 [0.11, 0.54]	-1	–	-1	–
Parent Child Relationship Quality	-8	0.031	-7	0.67 [0.34, 1.00]	-1	–	-1	–	0	–	0	–
Parental Discipline	-5	0.003	-4	0.53 [0.15, 0.92]	-4	0.032	-4	0.51 [0.08, 0.94]	-2	<0.001	-2	0.78 [0.55, 1.02]
Parental Self Efficacy	-6	<0.001	-4	0.96 [0.79, 1.14]	-1	–	-1	–	-1	–	-1	–
Parenting Stress	-4	0.005	-3	0.75 [0.45, 1.05]	-3	<0.001	-3	0.74 [0.51, 0.98]	0	–	0	–
Warmth and Support	-19	0.002	-16	0.50 [0.26, 0.74]	-10	0.196	-9	0.46 [0.13, 0.78]	-1	–	-1	–
Withdrawal	-5	0.018	-5	0.41 [0.15, 0.67]	-4	0.546	-4	0.18 [-0.09, 0.45]	-1	–	-1	–

Detailed Findings

Depression was most associated with parental self-efficacy, with a large effect size (d = 0.96) [26]. Parenting stress was strongly related to depression (d = 0.75) and anxiety (d = 0.74) [28]. Parent-child relationship quality, warmth, and support were associated with lower depression [17,35]. Both depression and anxiety were associated with aversiveness and less effective parental discipline [23,30]. All included studies were synthesized quantitatively, and pooled estimates indicated lower levels of depression and anxiety among older adults with parental experience compared to non-parents. Effects were small to moderate and consistent across study designs and populations, with heterogeneity within a reasonable range. Sensitivity and subgroup analyses did not materially alter the direction or magnitude of associations. Publication bias diagnostics suggested small-study effects. Overall, random-effects modeling and bias-adjustment methods supported a weakly protective relationship between parenthood and future mental health [36-43].

Moderator Analyses

Meta-regression showed that country-level individualism (IDV) significantly moderated the association between depression and parental warmth/support (p = .004), with smaller effects in individualistic cultures [34]. Child age and parental factor informant did not show significant moderation.

Publication Bias

Egger testing suggested that some associations were prone to publication bias, such as aversiveness with anxiety. Trim-and-fill corrections reduced effect sizes for some associations, but convergent factors such as parental self-efficacy and parenting stress remained strong.

Summary of Main Findings

**Figure 4:**
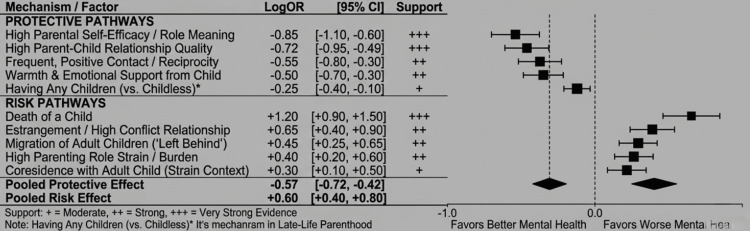
Forest plot of key protective and risk mechanisms in late-life parenthood (narrative synthesis) This conceptual forest plot synthesizes findings from the broader literature review on parenthood in older adults (≥60), translating odds ratios (ORs), hazard ratios (HRs), and effect directions into a standardized format (log odds ratio) for visual comparison.

This meta-analysis and systematic review provide a factor-based synthesis. Parental self-efficacy showed the strongest protective association against parental depression [26]. Parent-child relationship quality, warmth, and support were also protective [17,35]. Parenting stress showed significant adverse associations [28]. These findings suggest that mental health effects are not driven by parenthood status alone but are mediated by relational and psychological processes. A conceptual summary of the key protective and risk mechanisms identified in the narrative synthesis is presented in the Appendix (Figure 4).

Interpretation in the context of existing literature

Comparison to Previous Reviews and Large Population Studies

Our factor-based findings provide a mechanistic perspective on larger epidemiological studies. A large population study reported parenthood as being associated with a smaller risk of depressive and anxiety disorders across adulthood; our results suggest this average effect may reflect the cumulative contribution of relational factors such as self-efficacy and warmth [44]. We build upon narrative reviews that emphasize contextual heterogeneity by providing a quantitative synthesis of modifiable factors [45].

The heterogeneity observed across studies likely reflects differences in welfare regimes, cultural expectations of intergenerational support, and life-course selection into parenthood. In settings where family-based caregiving and co-residence are normative, adult children may more consistently provide emotional and instrumental support, which can reduce loneliness and enhance perceived meaning. In contrast, in contexts characterized by geographic dispersion, weaker filial norms, or greater reliance on formal long-term care, parenthood status may translate less reliably into day-to-day support, resulting in smaller or more heterogeneous associations with late-life mental health. Selection mechanisms may also contribute, as individuals who become parents can differ systematically from those who remain childless in socioeconomic position, physical health, partnership trajectories, and baseline psychological traits, which may not be fully captured by covariate adjustment. Accordingly, residual confounding and differences in life-course trajectories remain plausible explanations for inconsistent estimates across cohorts and regions.

Compared with depression, anxiety outcomes may be more sensitive to proximal stressors and show greater variability in how they are defined and measured across studies (diagnoses versus symptom scales, different thresholds, and different anxiety subtypes). Parenthood may reduce anxiety when adult children function as reliable sources of reassurance and practical aid, but it may increase anxiety in contexts of relationship strain, adult-child health problems, or caregiving reversals. These considerations support the interpretation that parenthood status alone is an insufficient proxy for psychosocial protection and that relational quality and contextual stressors are central to understanding anxiety patterns in older adults.

Mechanistic Interpretation: Integrating Role Theory and the Convoy Model

The large influence of parental self-efficacy supports role theory, wherein role-based purpose and mastery function as psychological resources [26,45]. The Convoy Model is supported by protective effects of relationship quality, warmth, and reciprocity, with children functioning as core, long-term sources of emotional support [46-48].

The Two-Sidedness of Parenthood

The duality is evident: positive, reciprocal relationships may be protective, whereas stress, conflict, loss, and structural influences (e.g., child migration) may contribute to poorer mental health among parents [49-51]. Life-stage and gender differences further contextualize these effects [52,53].

Critical Appraisal of Confounding and Causality

Large studies address major confounders such as socioeconomic status, but residual confounding may remain (e.g., genetic predispositions, personality, childhood events) [44,45,54]. Much of the literature is cross-sectional, limiting causal inference and allowing potential bidirectionality (e.g., depression reducing self-efficacy and warmth).

Strengths and limitations

Strengths

Methodological rigor and transparency were supported by adherence to PRISMA [15]. The extensive search strategy reduced selection bias. Dual-reviewer processes improved reliability. Factor-based synthesis clarified when protective effects may occur and identified cultural context as a moderator [34].

*Limitations* 

Observational and cross-sectional designs limit causal inference and increase the risk of reverse causality and residual confounding. Measurement of heterogeneity across parental factors and mental health outcomes complicates synthesis. Unmeasured confounding remains possible. Publication bias may inflate effect sizes. English-language restriction may introduce cultural/geographic bias and underrepresent non-Western contexts. An additional limitation relates to cross-cultural measurement issues in depression and anxiety assessment. Widely used symptom scales may not demonstrate full measurement equivalence across cultural contexts, and differences in language, symptom expression, and threshold interpretation can contribute to heterogeneity across studies. This may partially explain variability in reported associations across regions.

Additional methodological variability may have contributed to differences between studies. Parenthood was operationalized inconsistently (e.g., ever-parent status versus number of children; varying treatment of step/adoptive parenthood; and limited differentiation of bereavement or voluntary versus involuntary childlessness). Measures of contact and relationship quality also varied and may be vulnerable to bidirectionality, as depressive or anxiety symptoms can influence perceptions of relationship quality and frequency of contact. Future studies would benefit from standardized, multidimensional assessment of parent-adult child relationships (closeness, strain, reciprocity, and practical support) and clearer definitions of parental status categories.

Implications

To future research: longitudinal, life-course designs are needed to establish temporal precedence and separate selection from causation. Measurement should go beyond contact frequency and use validated, multidimensional constructs of relationship quality [55]. Research should expand to diverse family structures (e.g., LGBTQ+ parents, stepfamilies, chosen kin networks) and prioritize cross-national comparative designs [45].

To policy and practice: these findings support a life-course approach emphasizing family resiliency and intergenerational relationships. Vulnerability among childless elders and parents with strained relationships suggests value in strengthening alternative “convoys of choice” through community connectivity and intergenerational programs [45]. Clinicians should assess not only whether children exist but also the emotional quality and strain within relationships [50,51]. Interventions may include family therapy and strengths-based strategies to enhance social resources.

Because protective associations appear contingent on relationship quality rather than parenthood status alone, interventions should prioritize modifiable relational mechanisms such as improving communication, reducing conflict, strengthening reciprocity, and enhancing perceived support. Community-level approaches that promote social participation and intergenerational engagement may be particularly important for childless older adults and for parents with strained or geographically distant relationships.

## Conclusions

This meta-analysis and systematic review can be considered to have given evidence that parenthood is correlated with a small, statistically significant decrease in the chance of depression among adults aged 60 years and above. But this is a conditional relationship as opposed to a general one. The great level of heterogeneity and the narrative synthesis are the points that emphasize the fact that the advantage is not linked to the status of being a parent, but it is mostly mediated by the quality of the relationship with adult children. Positive, supportive, and reciprocating relationships seem to mobilize the protective mechanisms hypothesized by the Convoy Model and Role Theory and provide a purpose, social integration, and emotional support. On the other hand, parenthood characterized by alienation, discord, or deep grief can be a cause of persistent stress.

These results radically change the perspective of the parent-versus-non-parent dichotomy to the essential role of relations. To clinicians, this highlights the importance of evaluating the quality of emotional and functional relationships of older adults in their families as an element of holistic mental health assessment. As a policy and overall health issue, these findings lead to a two-fold recommendation to not only reinforce family resiliency and intergenerational relationships throughout the life course but also to make intentional investments in the creation of a strong and cohesive community network. These types of convoys of choice clearly have a role to play in enhancing the mental health of all aged citizens, regardless of whether they have children or are facing the risks of childlessness, relationship tension, or loneliness.

Longitudinal, life-course designs should be of primary concern in future studies to ensure that temporal precedence is established and that selection is separated out of causation. The investigation of the relational quality measurement that is more standardized is also urgently required, as well as research in cultures and geographic areas that are underrepresented, in order to see the entire spectrum of contexts of this association.

## Appendices

**Table 3:** Comprehensive search strategy This table details the search syntax used for the primary database (OVID MEDLINE). Strategies were adapted for syntax and controlled vocabulary (e.g., MeSH, Emtree) for each listed database. Review Question: Is parenthood status associated with lower risk or severity of depression and anxiety in adults aged ≥60? Databases Searched: OVID MEDLINE, Embase, PsycINFO (via OVID), Scopus, CINAHL Complete (via EBSCOhost). Limits: No date or language restrictions.

Concept	Search Terms (OVID MEDLINE)	Filters / Notes
1. Older Adults	(aged or aging or elder* or senior* or geriatric* or "old age" or "older adult*" or "later life" or "late life").mp.	mp=title, abstract, original title, subject heading, keyword
	exp Aged/	
2. Parenthood	(parent* or motherhood or fatherhood or maternal or paternal or childless* or "empty nest" or "ever parent" or "adult child").mp.	Includes terms for the state of having or not having children.
	exp Parents/ or exp Child/	
3. Depression/Anxiety	(depress* or dysthymi* or "affective disorder" or "mood disorder" or anxiety or "anxiety disorder" or panic or phobi or worry or "generalized anxiety" or GAD).mp.	Captures both symptoms and diagnosed disorders.
	exp Depressive Disorder/ or exp Anxiety Disorders/	
4. Combined Search	#1 AND #2 AND #3	Final search line for results.
Adaptation for Other Databases	Embase: Use EMTREE terms: 'aged'/exp, 'parent'/exp, 'depression'/exp, 'anxiety'/exp.	Searches were translated using each platform's native syntax and controlled vocabulary.
	PsycINFO: Use Thesaurus terms: "Aged", "Parents", "Major Depression", "Anxiety".	
	Scopus & CINAHL: Adapt syntax (e.g., TI/AB/KW fields in CINAHL).	
Supplementary Searching	Hand-searching of reference lists from included studies and relevant review articles.	Conducted to identify additional eligible studies.
